# Spatial and Temporal Trends of Access to Birthing Hospitals in Illinois, 2016–2021

**DOI:** 10.5888/pcd23.260064

**Published:** 2026-08-20

**Authors:** Mechelle D. Claridy, Barbara Chebet Keino, Lauren M. Ramsey, Elizabeth J. Conrey, Laurin Kasehagen, Jessica R. Meeker, Amanda C. Bennett

**Affiliations:** 1 Maternal and Child Health Epidemiology Program, Field Support Branch, Division of Reproductive Health, National Center for Chronic Disease Prevention and Health Promotion, Centers for Disease Control and Prevention, Atlanta, Georgia; 2 Epidemic Intelligence Service, Public Health Infrastructure Center, Division of Workforce Development, Centers for Disease Control and Prevention, Atlanta, Georgia; 3 HIV Surveillance Branch, Division of HIV Prevention, National Center for HIV, Viral Hepatitis, STD, and TB Prevention, Centers for Disease Control and Prevention, Atlanta, Georgia; 4 Emergency Preparedness and Response Team, Field Support Branch, Division of Reproductive Health, National Center for Chronic Disease Prevention and Health Promotion, Centers for Disease Control and Prevention, Atlanta, Georgia; 5 US Public Health Service Commissioned Corps, Rockville, Maryland

## Abstract

**Introduction:**

Changes in the availability of birthing hospitals may affect geographic access to obstetric care. Travel time to the nearest birthing hospital is a measurable indicator of access. We estimated changes in travel time in Illinois from 2016 to 2021 and described the distribution of populations living in census tracts with increased travel time.

**Methods:**

We used Illinois Department of Public Health hospital data (2016–2021), US Census population-weighted block group (PWBG) centroids, and American Community Survey data (2017–2021). We estimated driving time from PWBG centroids to the nearest in-state birthing hospital (driving time) by using ArcGIS network analysis and averaging to census tracts. We assessed changes overall and by rural–urban classification, race and ethnicity, and educational attainment.

**Results:**

The number of Illinois birthing hospitals decreased 16.9%, from 118 in 2016 to 98 in 2021. Average driving time increased from 12.1 to 13.2 minutes overall, from 9.5 to 10.4 minutes in urban areas, and from 24.1 to 25.9 minutes in rural areas. Overall, 18.3% of Illinois residents (2,288,738 of 12,502,452) lived in census tracts with increased driving time. Among residents living in rural areas, 23.7% lived in affected tracts, compared with 17.4% among those living in urban areas. Among non-Hispanic White residents in Illinois, 14.9% lived in affected tracts, compared with 32.1% of non-Hispanic Black residents. Among college graduates, 14.8% lived in affected tracts, compared with 23.3% of residents with less than a high school education.

**Conclusion:**

From 2016 to 2021, changes in the availability of birthing hospitals in Illinois coincided with modest increases in driving time and were not evenly distributed across populations. These findings provide a descriptive assessment of geographic access to obstetric care and may inform efforts to address differences in access.

**Summary**

**What is already known on this topic?**

Closures of obstetric units in the US have reduced geographic access to maternity care, particularly in rural areas, where hospitals are more sparsely distributed.

**What is added by this report?**

From 2016 to 2021 in Illinois, the number of birthing hospitals decreased, and average driving time from Illinois census tracts to the nearest in-state birthing hospital increased modestly. Approximately 1 in 5 residents lived in areas with increased travel time, with differences observed across rural–urban areas and population groups.

**What are the implications for public health practice?**

Monitoring geographic access to obstetric care can help identify areas and populations affected by changes in service availability and inform strategies to support access.

## Introduction

Access to obstetric care in the US has been affected by closures of birthing hospitals, contributing to changes in geographic access to care (1,2). Longer travel times to birthing hospitals may present barriers to timely care, particularly in emergency situations (3). Closures have been attributed to multiple factors, including financial constraints, workforce shortages, and declining birth volumes, particularly in rural areas (1,2). In 2022, nearly 6 million women of reproductive age lived in counties without a hospital providing obstetric services (2). Research has documented the emergence of areas with limited access to maternity care, often referred to as maternity care deserts, as well as differences in access between rural and urban communities (2–5). An important and measurable dimension of access is driving time to the nearest birthing hospital.

In this study, we defined a birthing hospital as a hospital that provides obstetric delivery services, consistent with state-level classifications. Illinois has a regionalized perinatal care system, established in 1976, with 10 administrative perinatal centers overseeing hospital networks across the state (6). These centers support care coordination, provider education, and maternal and neonatal transport services, particularly for high-risk pregnancies (7). Multiple birthing hospitals have closed in Illinois in recent years, affecting both urban and rural areas. Given the state’s mix of densely populated urban centers and geographically dispersed rural regions, changes in birthing hospital availability may correspond to changes in travel time to access care.

Travel time is a commonly used measure of geographic access to health care services. This study aimed to 1) examine average driving time and distance from Illinois census tracts to the nearest in-state birthing hospital in 2021 and assess changes from 2016 overall and by rural–urban classification and 2) assess the distribution of populations residing in census tracts with increased driving time overall and by rural–urban classification.

## Methods

### Data sources

We obtained data on Illinois census tract boundaries from the 2020 US Census Bureau’s decennial census. Census tracts are small geographic units, typically containing 1,200 to 8,000 people, and are commonly used to examine local population characteristics (8). Within census tracts, populations are further subdivided into block groups. We obtained data on population-weighted block group (PWBG) centroids, which represent the mean center of populations within block groups, from the 2020 United States Census (8).

We used Illinois Department of Public Health hospital data from 2016 to 2021 to identify birthing hospitals. Illinois birthing hospitals are designated as level 1 (able to care for uncomplicated maternal and newborn patients), level 2 or level 2 with extended neonatal capabilities (2E) (able to care for some maternal and newborn complications), or level 3 (able to care for critically ill maternal and newborn patients) (6). We considered hospitals designated as level 1 or higher at the end of each calendar year to have obstetric care services and to be birthing hospitals.

We used the 2016–2021 American Community Survey 5-year estimates to obtain census tract-level population estimates by race and ethnicity and educational attainment. Race and ethnicity were categorized as Hispanic/Latino (of any race), non-Hispanic American Indian and Alaska Native (AIAN), non-Hispanic Asian, non-Hispanic Black, non-Hispanic Native Hawaiian or Pacific Islander, non-Hispanic White, and non-Hispanic Other or multiple races. Educational attainment (the highest level of education an individual completed) was categorized as less than high school graduate, high school graduate, some college, or college graduate. This study was reviewed by the Centers for Disease Control and Prevention (CDC), deemed not research, and was conducted consistently with applicable federal law and CDC policy.

### Estimation of driving time and distance

The primary outcome was the average driving time (in minutes) and distance (in miles) from census tracts to the nearest birthing hospital in Illinois. We calculated driving times and driving distances between PWBG centroids and birthing hospitals using the “Find Nearest” network analysis tool in ArcGIS Online. The tool uses a road network dataset with driving-time impedance (ie, the degree of difficulty or resistance to travel) to estimate travel along available roadways, including local and major roads. Driving times represent modeled estimates under typical conditions and do not account for real-time traffic, weather, time of day, or individual behaviors. We did not include out-of-state birthing hospitals in the analysis.

For each year, we calculated driving time and distance from PWBG centroids to the nearest birthing hospital and tract-level averages as the unweighted mean of PWBG-level values within each census tract (9).

We classified PWBG centroids as rural or urban according to the 2020 US Census Bureau’s Urban Areas classification (8). We then classified census tracts as rural or urban based on whether most of the population (estimates from the 2021 American Community Survey 5-year survey) within the tract resided in rural or urban block groups.

Given that census tracts were measured at multiple time points and are spatially related, we conducted analyses as descriptive comparisons rather than inferential statistical tests. We summarized changes in driving time and distance from 2016 to 2021 overall and by rural–urban classification.

### Changes in driving time by population characteristics

We categorized census tracts according to change in average driving time from 2016 to 2021 as 1) no increase in driving time (including tracts with unchanged or decreased driving time) or 2) increase in driving time. Among tracts with increases, we further categorized the magnitude of change as less than 10 minutes or 10 minutes or more. We selected these cut points to support interpretability of changes in access; we also examined continuous measures to ensure that findings were not sensitive to categorization.

We calculated the proportion of Illinois residents within each population group (race and ethnicity and educational attainment) living in census tracts with increased driving time. We conducted analyses on the overall population and stratified by rural–urban classification. We conducted all analyses using SAS version 9.4 (SAS Institute Inc).

## Results

### Hospital closures

The number of Illinois birthing hospitals decreased from 118 in 2016 to 98 in 2021 (17% decrease). Level 1 and level 2/2E birthing hospitals decreased from 93 to 72 (23% decrease), while level 3 birthing hospitals increased from 25 to 26 (4% increase), reflecting the reclassification of one hospital from level 2E to level 3.

### Driving time and distance

**Figure 1:**
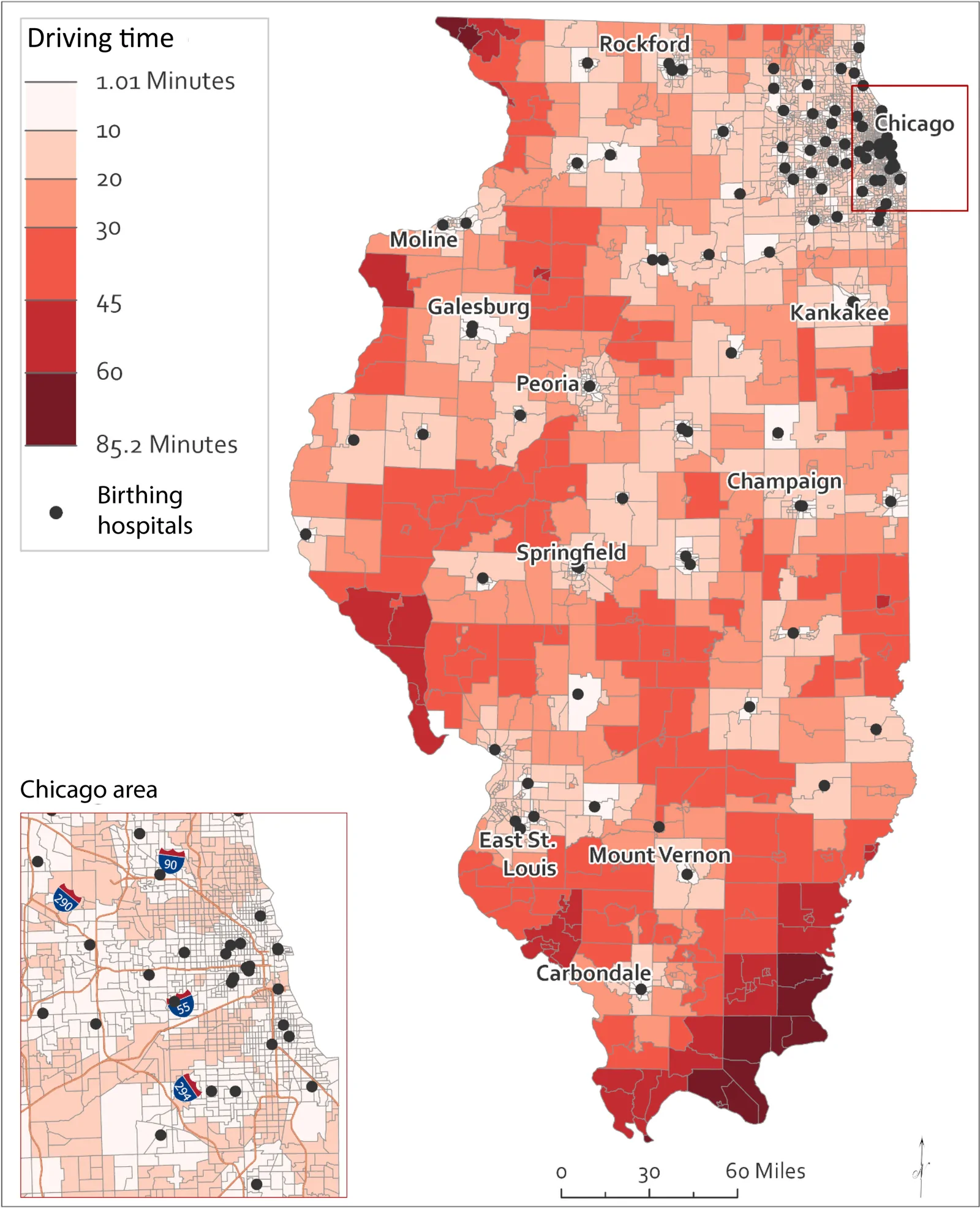
Average driving time to the nearest birthing hospital in Illinois per census tract in 2021.

In 2021, the average driving time from Illinois census tracts to the nearest in-state birthing hospital was 13.2 minutes, and the average driving distance was 7.8 miles (Table 1). Driving time to the nearest in-state birthing hospital was not evenly distributed across the state and varied even within large metropolitan areas (Figure 1). Travel times were generally shorter in urban areas than in rural regions. Census tracts located near state borders, particularly in western and southeastern Illinois, had longer driving times to the nearest in-state birthing hospital, reaching up to 85 minutes (Figure 1).

**Table 1:** Change in Average Driving Time and Distance to the Nearest Birthing Hospital in Illinois, Overall and by Rural–Urban Classification, 2016–2021^a^

Type	Average driving time (95% CL), min			Average driving distance (95% CL), mi		
	2016	2021	Change	2016	2021	Change
Overall	12.1 (11.9–12.3)	13.2 (13.0–13.4)	+1.1	7.0 (6.9–7.2)	7.8 (7.6–8.0)	+0.8
Urban tracts	9.5 (9.4−9.7)	10.4 (10.3–10.6)	+0.9	4.5 (4.4–4.6)	5.1 (5.0–5.2)	+0.6
Rural tracts	24.1 (23.6–24.7)	25.9 (25.3–26.5)	+1.8	18.8 (18.2–19.3)	20.2 (19.6–20.7)	+1.4

Abbreviation: CL, confidence limit.

a Driving time and distance were estimated from population-weighted block group centroids to the nearest in-state birthing hospital by using ArcGIS network analysis and averaging to census tracts.

### Changes in driving time

**Figure 2:**
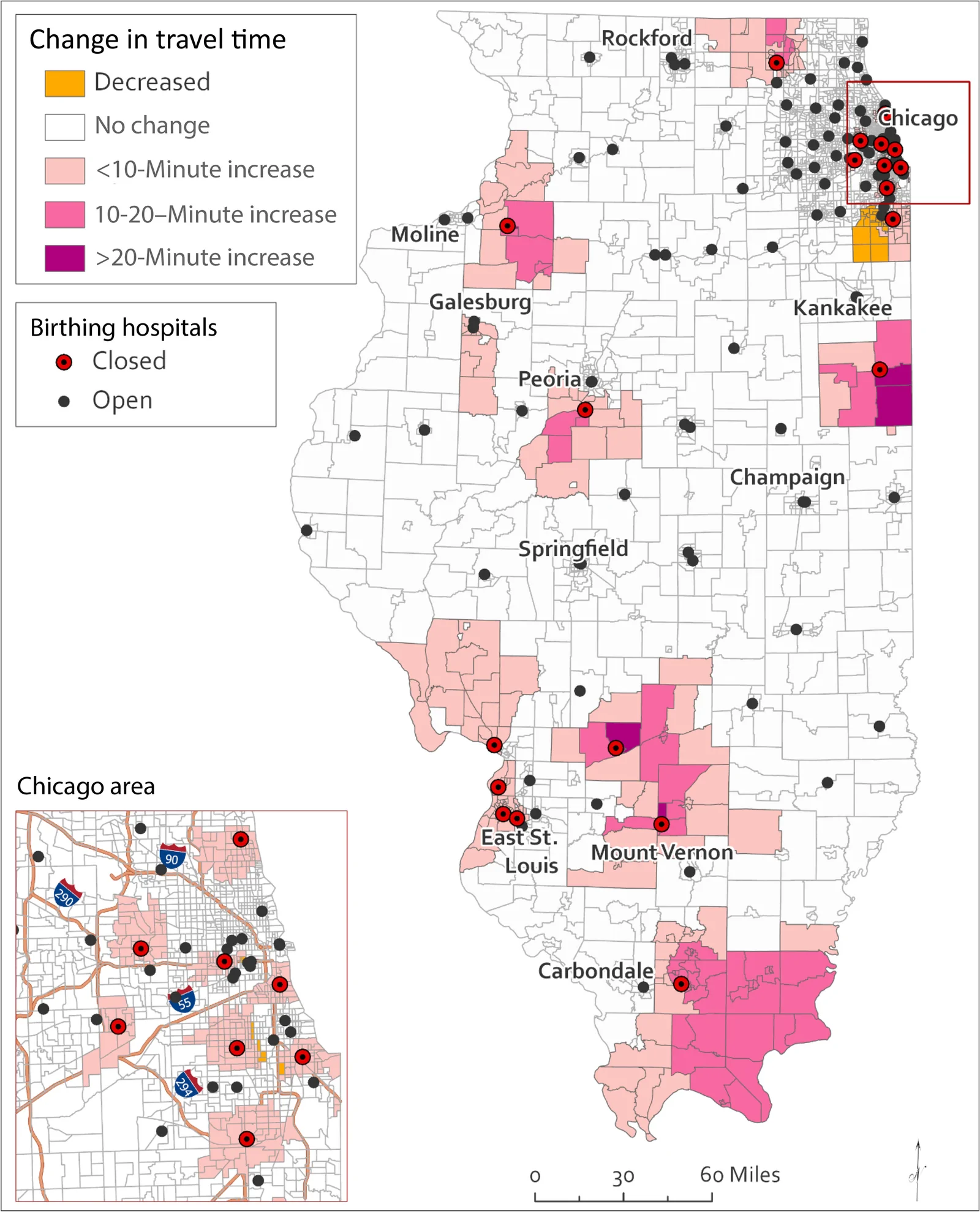
Difference in average driving time from census tracts in Illinois to the nearest in-state birthing hospital per census tract, 2016 to 2021.

From 2016 to 2021, average driving time to the nearest in-state birthing hospital increased from 12.1 to 13.2 minutes overall, and average driving distance increased from 7.0 to 7.8 miles. In urban areas, average driving time increased from 9.5 to 10.4 minutes (from 4.5 to 5.1 miles), while in rural areas it increased from 24.1 to 25.9 minutes (from 18.8 to 20.2 miles) (Table 1). Most census tracts experienced no change in driving time, and a small number experienced decreases (Figure 2). Increases in driving time were not evenly distributed across the state but were concentrated in some areas, including areas near Chicago, East St. Louis, Kankakee, Carbondale, and Moline (Figure 2). In rural regions, particularly in southern and western Illinois, increases tended to affect larger, more contiguous areas, whereas in urban areas, increases were more localized (Figure 2). When examined as a continuous measure, the distribution of driving time changes showed similar patterns; most increases were small, supporting the use of categorical thresholds for interpretability.

### Populations experiencing increased driving time

Approximately 1 in 5 (18.3%) Illinois residents lived in a census tract that experienced an increase in driving time to the nearest in-state birthing hospital from 2016 to 2021 (Table 2). Among residents living in affected tracts, 86.5% experienced increases of less than 10 minutes, while 13.5% experienced increases of 10 minutes or more.

**Table 2:** Residents With Increased Driving Time to the Nearest Birthing Hospital in Illinois, Overall and by Rural–Urban Classification, 2016–2021^a^

Population group	Population, no.	Any increase, no. (%)	Increase <10 min, no. (%)^b^	Increase ≥10 min, n (%)^b^
All Illinois residents	12,502,452	2,288,738 (18.3)	1,978,986 (86.5)	309,752 (13.5)
Urban residents	10,755,651	1,875,023 (17.4)	1,712,453 (91.3)	162,570 (8.7)
Rural residents	1,746,801	413,715 (23.7)	266,533 (64.4)	147,182 (35.6)

a Population estimates are from the 2021 American Community Survey.

b Among residents in census tracts with increased driving time.

Among residents living in rural areas, 23.7% lived in affected tracts, compared with 17.4% among those living in urban areas. Among rural residents living in affected tracts, 35.6% experienced increases of 10 minutes or more, compared with 8.7% among urban residents living in affected tracts (Table 2).

### Increases by racial and ethnic characteristics

The proportion of Illinois residents living in census tracts with increased driving time varied by race and ethnicity, ranging from 13.8% among non-Hispanic Asian residents to 32.1% among non-Hispanic Black residents (Table 3).

**Table 3:** Race and Ethnicity and Educational Attainment of Residents, by Change in Driving Time to the Nearest Birthing Hospital in Illinois, Overall and by Rural–Urban Classification, 2016–2021^a^

Population group	All residents		Urban		Rural	
	Population, no.	Increase, no. (%)	Population, no.	Increase, no. (%)	Population, no.	Increase, no. (%)
**All residents**	12,502,452	2,288,738 (18.3)	10,755,651	1,875,023 (17.4)	1,746,801	413,715 (23.7)
**Race and ethnicity**						
Hispanic/Latino	2,243,275	461,197 (20.6)	2,174,382	441,847 (20.3)	68,893	19,350 (28.1)
Non-Hispanic American Indian and Alaska Native	10,717	2,113 (19.7)	9,067	1,590 (17.5)	1,650	523 (31.7)
Non-Hispanic Asian	720,091	99,534 (13.8)	707,180	97,884 (13.8)	12,911	1,650 (12.8)
Non-Hispanic Black	1,762,122	565,213 (32.1)	1,716,919	550,555 (32.1)	45,203	14,658 (32.4)
Non-Hispanic Native Hawaiian/Pacific Islander	2,940	473 (16.1)	2,310	292 (12.6)	630	181 (28.7)
Non-Hispanic White	7,728,906	1,153,742 (14.9)	6,113,888	776,993 (12.7)	1,615,018	376,749 (23.3)
Other or multiple races	34,401	6,466 (18.8)	31,905	5,862 (18.4)	2,496	604 (24.2)
**Educational attainment**						
Less than high school graduate	885,321	206,186 (23.3)	785,873	179,020 (22.8)	99,448	27,166 (27.3)
High school graduate	2,226,182	472,094 (21.2)	1,800,275	368,453 (20.5)	425,907	103,641 (24.3)
Some college	1,761,534	354,179 (20.1)	1,474,881	283,031 (19.2)	286,653	71,148 (24.8)
College graduate	3,891,841	577,686 (14.8)	3,451,647	479,747 (13.9)	440,194	97,939 (22.2)

a Population estimates are from the 2021 American Community Survey.

For most racial and ethnic groups, the proportion living in affected tracts was higher among rural residents than urban residents. This rural–urban gap was largest among non-Hispanic AIAN residents, with 31.7% of all rural AIAN residents living in affected tracts compared with 17.5% of all urban AIAN residents. We observed a similar rural–urban gap among non-Hispanic Native Hawaiian or Pacific Islander residents (28.7% rural vs 12.6% urban), Hispanic residents (28.1% rural vs 20.3% urban), and non-Hispanic White residents (23.3% rural vs 12.7% urban).

Non-Hispanic Black and non-Hispanic Asian residents did not follow this pattern. Among all non-Hispanic Black residents in Illinois, 32.1% lived in affected tracts, the highest proportion of any group, and this proportion was nearly identical among those living in rural (32.4%) and urban (32.1%) areas. Among all non-Hispanic Asian residents in Illinois, 13.8% lived in affected tracts, the lowest proportion of any group, with similar proportions among rural (12.8%) and urban (13.8%) residents (Table 3).

### Increases by educational attainment

The proportion of Illinois residents living in census tracts with increased driving time differed by educational attainment. Among residents in Illinois with less than a high school education, 23.3% lived in affected tracts, compared with 14.8% of all college graduates (Table 3).

We observed a similar pattern across rural and urban areas. In both settings, higher proportions of residents with lower levels of education lived in affected tracts compared with those with higher levels of education. Although proportions were higher in rural areas across all education groups, the overall gradient by educational attainment remained consistent. For example, 27.3% of rural residents with less than a high school education lived in affected tracts, compared with 22.8% of urban residents with less than a high school education; similarly, 22.2% of rural college graduates lived in affected tracts, compared with 13.9% of urban college graduates in Illinois (Table 3).

## Discussion

From 2016 to 2021, the number of birthing hospitals in Illinois decreased, while average driving time to the nearest in-state birthing hospital increased modestly at the statewide level. Approximately 1 in 5 Illinois residents lived in census tracts that experienced an increase in driving time, although most increases were small. However, these changes were not evenly distributed across geographic areas or population groups. Although most affected census tracts experienced increases in driving time of less than 10 minutes, some census tracts experienced increases of 20 minutes or more, particularly in rural regions where facilities are more sparsely distributed. These findings underscore the importance of examining geographic variation in access to obstetric care.

Our findings are consistent with other studies that showed that driving time or distance increased in the context of obstetric unit closures (4,5,10). One study found that driving distance to access intrapartum care increased by an average of 29 additional miles when 7.2% of rural hospitals closed their obstetric units (5). These obstetric units were smaller, more likely to be privately owned, and located in communities with lower family income, fewer obstetricians, and fewer family physicians compared with obstetric units that did not close (5). Together, these findings suggest that reductions in local service availability can translate into measurable changes in geographic access.

In Illinois, we observed differences in driving time changes between rural and urban census tracts. Rural census tracts more frequently experienced larger increases in average driving time, consistent with the broader pattern observed nationally. This likely reflects the lower density of facilities in rural areas, where closures or service changes can result in greater increases in travel distance compared with urban settings. Prior studies showed that closures in rural counties were associated with shifts in where births occur and changes in use of prenatal care (4,11), highlighting the importance of geographic access in shaping patterns of care delivery.

Differences in the proportion of residents living in census tracts with increased driving time varied across population groups. Consistent with the rural–urban pattern described above, for most racial and ethnic groups, a higher proportion of residents living in rural areas lived in affected tracts compared with those living in urban areas. This rural–urban gap was particularly pronounced among non-Hispanic AIAN, non-Hispanic Native Hawaiian or Pacific Islander, Hispanic, and non-Hispanic White residents.

In contrast, non-Hispanic Black and non-Hispanic Asian residents did not follow this pattern. Among non-Hispanic Black residents in Illinois, the proportion living in affected tracts was similarly high in both rural and urban areas, representing the highest proportion of any group. Among non-Hispanic Asian residents, the proportion was similarly low across both settings, representing the lowest proportion of any group. These patterns suggest that the distribution of changes in geographic access differed across groups. For most populations, differences in geographic access were driven primarily by rural residence and greater distances between facilities. For non-Hispanic Black residents, however, the consistently high proportion across both rural and urban areas indicates that residents were more likely to live in census tracts located near areas where birthing hospital availability changed, regardless of geographic setting.

We observed a similar pattern by educational attainment. Residents with lower levels of education were more likely to live in census tracts with increased driving time than those with higher levels of education, and this gradient was consistent across both rural and urban areas. As with differences in racial and ethnic patterns, these differences likely reflect the geographic distribution of populations relative to where changes in access occurred, rather than individual-level risk.

Approaches to counteract potential negative consequences of birthing hospital closures include investing in health care infrastructure, including incentives to retain clinicians in areas experiencing such closures (12). Strengthening systems that support transport of pregnant women to hospitals with obstetric services, such as equipment and consultation resources for emergency medical services, can help ensure timely access when facilities are farther away (13). Expanding the obstetric care workforce by supporting midwifery and doula services may increase access to appropriate care, reflect the lived experiences of diverse communities, and reduce associated costs (1,14,15). Telehealth programs, including virtual prenatal visits and remote patient monitoring, offer a complementary approach for reducing the burden of travel for outpatient care in areas with limited access (16). Furthermore, both the National Rural Health Association and the American College of Obstetricians and Gynecologists highlight the importance of regionalized perinatal centers for providing rural and low-resource hospitals with ready access to telemedicine consultation, referral, and outreach education (13,17).

In Illinois, these approaches are supported in part through the state’s regionalized perinatal care system. The Illinois Department of Public Health continues to support regionalized perinatal centers to provide necessary education, outreach, consultation, and transport services with network hospitals, including those without birthing services (11,18,19). These centers also play a key role in quality improvement initiatives and provider support and may offer opportunities to expand obstetric training and simulation for rural health care providers in hospital emergency departments (18).

### Limitations and strengths

This study has several limitations. Driving time is a commonly used measure of potential access to health care services. Our study estimated driving time from population-weighted block group centroids rather than from individual addresses, which may not capture variation in travel patterns. Estimates were generated by using a routing algorithm and did not account for real-time traffic, weather conditions, or time-of-day variation. We assumed that individuals seek care at the nearest birthing hospital; however, actual hospital use may differ based on factors such as insurance coverage, provider networks, physician affiliations, and patient preferences. As a result, estimated driving times in this study reflect potential geographic access rather than realized access, and may not fully capture the care-seeking experiences of all populations, particularly in urban areas where multiple hospital options are available. The study also did not assess use of care, patient preferences, or health outcomes. In addition, out-of-state facilities were not included, which may have resulted in an overestimate of travel times near state borders. Finally, the analysis was not restricted to women of reproductive age and did not examine the causes of hospital closures.

Despite these limitations, this study has several strengths. We used Illinois Department of Public Health data to identify birthing hospitals and American Community Survey data to characterize population distributions. Our use of population-weighted block group centroids, rather than tract-level centroids, improves geographic precision. Additionally, GIS-based network analysis allowed for estimation of driving time and distance along road networks, providing more precise estimates of geographic access than straight-line distance measures, which are commonly used in similar analyses but may misrepresent actual travel time along available roadways.

Future work could examine hospital-seeking patterns and how individuals choose facilities when multiple options are available. Additional analyses could explore access across different levels of perinatal care and assess socioeconomic gradients in geographic access using area-level measures of income or deprivation. Understanding how changes in service availability intersect with population distribution will be important for planning and maintaining access to obstetric care.

### Conclusion

From 2016 to 2021, the number of birthing hospitals in Illinois decreased, and average driving time to the nearest in-state birthing hospital increased modestly. Approximately 1 in 5 Illinois residents lived in census tracts where driving time increased, although most increases were small. These changes were not evenly distributed across geographic areas or population groups, with larger increases concentrated in rural areas and higher proportions of some populations living in affected tracts. These findings provide a descriptive assessment of changes in geographic access to obstetric care. Monitoring how changes in service availability intersect with population distribution may help inform strategies to support access, particularly in areas where travel times are increasing.
